# Comparison between Collagen and Lidocaine Intramuscular Injections in Terms of Their Efficiency in Decreasing Myofascial Pain within Masseter Muscles: A Randomized, Single-Blind Controlled Trial

**DOI:** 10.1155/2018/8261090

**Published:** 2018-06-03

**Authors:** Aleksandra Nitecka-Buchta, Karolina Walczynska-Dragon, Jolanta Batko-Kapustecka, Mieszko Wieckiewicz

**Affiliations:** ^1^Department of Temporomandibular Disorders, Unit SMDZ in Zabrze, Medical University of Silesia in Katowice, Traugutta Sq. 2, 41-800 Zabrze, Poland; ^2^Department of Experimental Dentistry, Faculty of Dentistry, Wroclaw Medical University, 26 Krakowska St., 50-425 Wroclaw, Poland

## Abstract

**Background and Objective:**

A novel option for myofascial pain (MFP) management and muscle regeneration is intramuscular collagen injections. The aim of the study was to evaluate the efficiency of intramuscular injections of collagen and lidocaine in decreasing MFP within masseter muscles.

**Methods:**

Myofascial pain within masseter muscles was diagnosed on the basis of the Diagnostic Criteria for Temporomandibular Disorders (II.1.A. 2 and 3). A total of 43 patients with diagnosed MFP within masseter muscles were enrolled to the study (17 male and 26 female, 40 ± 3.8 years old) and randomly divided into three groups. The first group received injections using 2 ml of collagen MD Muscle (Guna), the second group received 2 ml of 2% lidocaine without a vasoconstrictor, and the third group 2 ml of saline as a control (0.9% NaCl). All patients received repeated injections at one-week intervals (days 0 and 7). The visual analogue scale was used to determine pain intensity changes during each follow-up visit (days 0, 7, and 14) in each group. The masseter muscle activity was measured on each visit (days 0, 7, and 14) with surface electromyography (sEMG) (Neurobit Optima 4, Neurobit Systems).

**Results:**

We found that sEMG masseter muscle activity was significantly decreased in Group I (59.2%), less in Group II (39.3%), and least in Group III (14%). Pain intensity reduction was 53.75% in Group I, 25% in Group II, and 20.1% in Group III.

**Conclusions:**

The study confirmed that intramuscular injection of collagen is a more efficient method for reducing myofascial pain within masseter muscles than intramuscular injection of lidocaine.

## 1. Introduction

Myofascial pain within masticatory muscles is a popular muscle disorder among patients attending dental practitioners [1–3]. Mental status and bruxism may lead to excessive muscle effort and development of muscle pain [4–7]. The main syndrome of myofascial pain is a trigger point, which is a hard, palpable, localized nodule, painful on compression [8]. Myofascial pain is a symptom of muscle damage. Muscle regeneration is similar to muscle embryonic cell development.

Muscle injury can occur as a result of disease (dystrophy), contact with miotoxins, trauma, contusion, ischemia, temperature, and excessive muscle contraction [9]. Eccentric muscle contraction results in muscle damage and inflammation, resulting in muscle collagen accumulation, and occurs during the repair process of exercise-induced muscle injury [10]. Mechanical stress and cryolesions also induce collagen accumulation and production. During mechanical damage to muscles, sarcomere myofilaments are disrupted, the sarcolemma is damaged and fibers disintegrate [9]. After the muscle damage, interleukin-6 is released, and it induces fibroblasts to produce collagen [11, 12]. During muscle regeneration, stem cells proliferate and undergo differentiation into myoblast cells [13]. Simons' integrated hypothesis postulates energy crisis as the reason for the initial sarcomere contracture, which leads to increased metabolism and decreased capillary blood circulation [14]. The result is local hypoxia, muscle damage, and inflammatory mediators releasing, for example, catecholamines, neuropeptides, and cytokines. Then, muscle inflammation, persistent pain, and myofascial tenderness begin. Contraction knots are formed, as an effect of local injury, ischemia, and fiber lock. The blood flow around and within the trigger point is diminished. High-resistance and retrograde diastolic blood flow in the trigger point have been observed [14]. Vascular resistance is caused by musculature contracture and vessel compression. The effect is pain, tenderness, and nodularity of muscle tissue. Järvholm et al. have found that intramuscular pressure in trigger points decreased local blood flow and caused local ischemia [15]. Many trigger points localized together form myogelosis, where the level of oxygen is extremely low. In this mechanism, the level of ATP (adenosine triphosphate) is decreased. ATP is necessary for breaking the bonds between muscle myofilaments after muscle contracture. A low level of oxygen is a potent factor for bradykinin release [14]. Current approaches for trigger point management are needling, injections, and deep massage.

Lengthening contractions or endurance training may cause skeletal muscle damage, especially to the extracellular matrix (ECM) and muscle fibers. Collagen synthesis in muscle tissue, after damage, is elevated for 3 days [16]. Procollagen is synthesized in the endoplasmic reticulum and is extruded into the ECM. Premature collagen (tropocollagen) is then altered into the matured collagen protein. ECM is essential in muscle cell development and regeneration, and it is an important cell surrounding, which coordinates cell behavior and communication [17]. Interactions between muscle cells and ECM build a very important network in tissues undergoing mechanical stress. The lack of collagen in ECM is a reason for inappropriate muscle regeneration and muscle dystrophies. The lower the number of newly formed microfibers, the fewer the cross-sectional connections and the lower the produced muscle mass [18]. Collagen is strictly needed for proper muscle regeneration. Collagen decreases apoptosis and increases myoblast proliferation [18]. The extracellular matrix is also necessary for growth factors (PDGF and TGF*β*s) which regulate the process of stem cell proliferation and differentiation. During healing after injury, ECM is remodeled. Undesired substitutions occur, when fibrotic, connective tissue substitutes for muscle cells. Excessive production of fibrillar collagen can produce a scar, instead of newly formed muscle tissue. In the beginning of the regeneration process, a thick collagen network is formed to locate myogenic cells [18]. Collagen extrusion is mainly performed by interstitial fibroblasts.

Muscle elastic modulus (*E* = 12 kPa) may increase (*E* > 18 kPa) after the muscle injury, during the regeneration phase, because of the higher muscle stiffness and collagen network organization [18]. In chronic temporomandibular disorders, we can observe a reorganization of muscle activity resulting in poor muscle function [19].

Muscle regeneration is performed by stem cells. Myogenic cells are located under the basal lamina surrounding myofibers. Muscle-specific stem cells—satellite cells—precursors of mature myofibers, are responsible for skeletal muscle regeneration after repeated injuries [20]. Stem cells are regulated by collagen VI: biochemical signals, promoting proliferation, and differentiation of newly formed muscle cells.

Collagen is a molecule in ECM that plays an important role in building the base membrane of the myofiber endomysium in skeletal muscles [21]. Collagen is a major protein in ECM of skeletal muscles that builds networks and also present in the nervous system (in endo, peri, and epineurium of Schwann cells) and maintains proper nerve myelination [22, 23]. Collagen is provided to the muscles by interstitial fibroblast cells. Fibroblasts synthetize collagen I and collagen III at different ratios during muscle regeneration. Cultured fibroblasts secrete and deposit collagen VI with beneficial effects on muscle stiffness. Fibroblasts are the main source of collagen and could become an attractive option for medical therapy in the future. Collagen also provides biochemical signals for satellite cells to proliferate into myocells [18]. It is the main component of ECM, needed for muscle regeneration. Excess collagen production can result in cicatrization [24]. Lehto et al. analyzed collagen synthesis in gastrocnemius muscle in rats [25]. 14C-labeled proline was administered intraperitoneally to animal calves. The radioactivity of muscle probes was measured by liquid scintillation spectrophotometry. The uptake of labeled collagen and glycosaminoglycans showed the exact regeneration period: between 10 and 14 days after an injury. The uptake decreased after 21 days post injury. A collagen matrix is injected to guide muscle cell regeneration and differentiation.

There are three phases of muscle regeneration: myofiber breakdown and inflammation; stem cell activation and proliferation; and differentiation into new myofibers [26]. Muscle regeneration can form either a functionally efficient muscle contractile system or a scar [27, 28]. First, necrosis takes place and myofibers are disrupted; the blood level of muscle protein is increased (creatine kinase and troponin). The first inflammatory cells in injured muscle are neutrophils, as soon as 1–6 h after the muscle damage [29, 30]. The next group of inflammatory cells is macrophages that appear in injured tissue after 48 h. The necessary condition for muscle regeneration is blood supply with a bloodstream. Revascularization is modulated by many endocrine factors, for example, the fibroblast growth factor (FGF), which has angiogenic properties. Transforming growth factor-beta (TGF*β*s) stimulates collagen production, proteoglycans, fibronectin, and ECM protein production and angiogenesis [25]. The platelet-derived growth factor (PDGF) also influences angiogenesis *in vivo*.

Lidocainum hydrochloricum 2% is used as a popular analgesic drug in dentistry and cardiology as an antiarrhythmic drug. The mechanism of action is one where sodium channels are blocked causing a decrease in the heart rhythm rate. Neurons cannot send signals to the central nervous system. This was discovered in 1946, and since then, it has been one of the most popular and essential drugs in medicine. It is used for infiltration, blocks, and surface tissue anesthesia. Lidocaine has a very fast onset of action: approximately 1.5 min. It is often used in combination with adrenaline to prolong the effect of anesthesia. In trigger point therapy, it is used without vasoconstrictor agents, because of the risk of ischemic necrosis. The length of analgesia duration is about 30 min to 3 hours. Lidocaine can also be used as an inhalation drug to prevent coughing, especially during intubation. Some patients can be unresponsive to lidocaine, for example, those with Ehlers–Danlos syndrome [31].

The aim of the study was to evaluate the efficiency of intramuscular injections of collagen and lidocaine in reducing MFP within masseter muscles.

## 2. Materials and Methods

### 2.1. Study Participants

Within a group of 102 Caucasian patients who had been referred to the Department of Temporomandibular Disorders at the Medical University of Silesia in Katowice, Poland, the principal investigator (ANB) found 50 with MFP within masseter muscles who were eligible and included in this trial.

The inclusion criteria were the following:

1. Age ≥18 and ≤80
2. Presence of myofascial pain and myofascial pain with referral within masseter muscles according to the Diagnostic Criteria for Temporomandibular Disorders (DC/TMD) (II.1.A. 2 and 3) [32]
3. Presence of trigger points within masseter muscles under palpation (latent or active)
4. Patients' agreement for taking part into the research study.

The exclusion criteria were the following:

1. Patients undergoing orthodontic treatment
2. Patients being treated with or addicted to analgesic drugs and/or drugs that affect muscle function
3. Patients after traumas to the head and neck region in the previous 2 years
4. Edentulous patients and patients with unsupported occlusal contacts in the lateral region of the occlusal arches
5. Patients being treated by neurologist for neurological disorders and/or neuropathic pain and/or headache
6. Patients after radiotherapy
7. Pain of dental origin
8. Pregnancy or lactation
9. Presence of malignancy
10. Presence of severe mental disorders
11. Drug and/or alcohol addiction
12. Presence of contraindications for injection therapy
13. Patients with needle phobia
14. Presence of hypersensitivity to substances to be used in the study.

This study was approved by the Bioethical Committee of the Medical University of Silesia in Katowice, Poland (KNW/0022/KB1/61/I/15), and retrospectively registered at ClinicalTrials.gov NCT03323567 (27 October 2017). The study was performed in accordance with the Declaration of Helsinki as well as the International Conference on Harmonisation: Guidelines for Good Clinical Practice. All included patients gave their consent to participate in the study and received verbal and written information describing the trial.

### 2.2. Study Protocol

This randomized, controlled, single-blind, three-arm trial followed the consolidated standards of reporting trials (CONSORT) statement [33] and was performed between 10 January 2016 and 12 December 2017 in the Department of Temporomandibular Disorders at the Medical University of Silesia in Katowice, Poland. The patients were divided randomly into three groups: Collagen (Group I, *n*=18), Lidocaine (Group II, *n*=15), and Saline (Group III, *n*=17). The randomization was carried out by a researcher who was not involved in the qualification of patients, conduct of interventions, or collection of data (MW). After allocation, 7 patients declined to participate. Consequently, the groups were structured as follows: Group I, *n*=15, 5 males, 10 females, mean age 37.2 ± 4.97; Group II, *n*=13, 5 males, 8 females, mean age 42.8 ± 0.98; and Group III, *n*=15, 7 males, 8 females, mean age 40.3 ± 1.18. Patients were not informed what substance they would be injected. The injections were performed by a principal investigator (ANB) who knew what substance she was administering.

**Figure 1:**
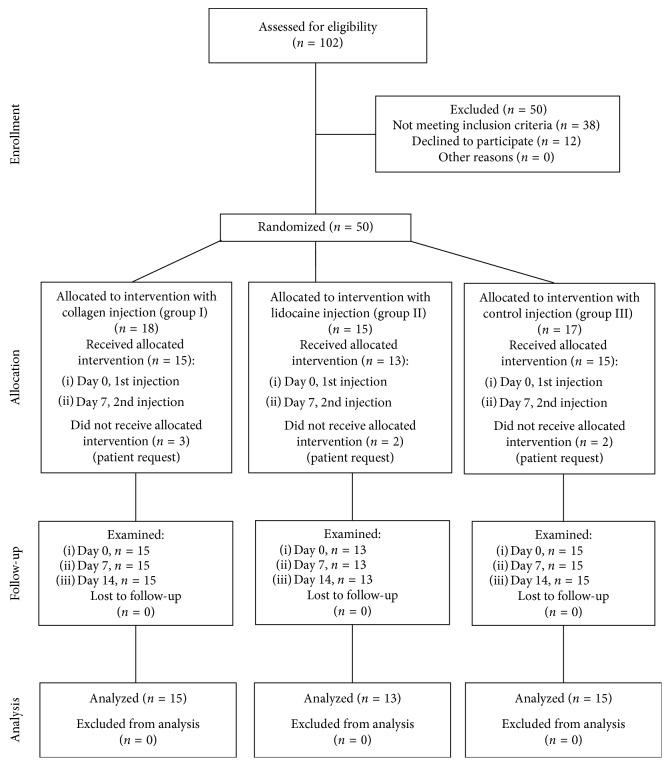
CONSORT three-arm diagram showing the flow of participants through each stage of the presented randomized controlled trial.

The trial consisted of four visits: (1) screening for study participation and inclusion, (2) first injection of study substances (baseline), (3) 1st follow-up and second injection of study substances, and (4) 2nd follow-up. The period between visits 2, 3, and 4 was one week (0, 7, and 14 days) (Figure 1).

The activities undertaken by the investigators during the trial are presented in Table 1.

### 2.3. Treatment

Group I was injected into the masseter trigger points using 2 ml of Collagen MD Muscle (Guna, Italy), Group II 2 ml of 2% Lidocaine (Lignocainum hydrochloricum WZF, Polfa Warsaw, Poland) without vasoconstrictor, and Group III 2 ml of saline as a control (0.9% NaCl) at 2nd and 3rd visits. In all groups, disposable syringes (2 ml) and needles (0.4 × 19 mm) were used for injections. During the intervention, trigger points within masseter muscles were identified with palpation of the masseter muscle, and each group was injected with the same amount of the appropriate substance (2 ml) into the trigger point structure. Injections were deposited approximately 1–1.5 cm under the skin surface. In 40 patients, the injections were unilateral and in 3 patients, bilateral in two masseter muscles with the same substance (2 subjects in Group I and 1 subject in Group II).

### 2.4. Treatment Outcome Measures

To measure treatment outcome, a surface electromyography (sEMG) and visual analogue scale (VAS) were used at the 2nd, 3rd, and 4th visits with one week breaks between visits (0, 7, and 14 days). For the assessment of masseter muscle activity, a surface electromyography was performed with a Neurobit Optima device (Neurobit Systems, Poland). The rest values for masseter muscle were measured for both sides. Muscle activity in the form of surface electromyography data was measured with 5 electrodes positioned bilaterally: in the origin region on the zygomatic arch and maxillary process of the zygomatic bone and in the insertion region on the angle and lateral surface of the mandible ramus. Two electrodes were positioned at each side of the patients head and one, a reference electrode, on the patient's neck. The patient remained seated on a dental chair, keeping his or her mandible in a resting, comfortable, and relaxed position, without tooth contact. The electromyographic evaluation was performed after cleaning the skin surface with cotton pads and an alcohol solution (Octenisept, Schulke, Germany). Electrodes were fixed on the skin covering the masseter muscle and on the patient's neck with a self-adhesive gel. The patient was asked to perform an isometric contraction of the masseter muscles to find the best place for electrode fixation. A 0–10 visual analogue scale with the endpoints marked “no pain” (0) and “worst experienced pain” (10) was used to evaluate the effectiveness in pain reduction of the substances studied. Pain evaluation using VAS and surface electromyography was performed by two investigators (JBK and KWD) and muscle injections were performed by the other investigator (ANB).

### 2.5. Sample Size Estimation

Normal distribution of VAS values was assumed. With the division into three groups, the analysis of variance for repeated measurements was planned, with equal sized groups. The power to achieve was 0.9 with the significance level set to 0.05.

Additional assumptions were the following:

1. Expected VAS values in individual research groups and subsequent measurements (Table 2).
2. Standard deviation for all measurements was SD = 1.5.
3. For the correlation matrix, the LEAR (linear exponent AR (1)) model was adopted, with base correlation set to 0.85 and correlation decay rate equal to 1.

The total number of subjects needed was 36, given the above assumptions; thus, the minimum number of subjects per group was 12. Sample size estimation was performed by using SAS, version 9.4 (SAS Institute Inc., Cary, NC).

### 2.6. Randomization and Blinding

Patients who met the inclusion criteria were randomized by computer-generated simple randomization into one of the following groups: Collagen (Group I, *n*=18), Lidocaine (Group II, *n*=15), and Saline (Group III, *n*=17). MW conducted the randomization and prepared the list of interventions by enrolment numbers. ANB administered the injections, according to the list. Patients and members of the study group (ANB, JBK, and KWD, who performed and collected pain intensity using VAS and muscle activity using surface EMG) were blinded for allocation and treatment.

### 2.7. Statistical Analysis

A one-way repeated measures analysis of variance was carried out. To verify the assumptions of the method in all groups, the analysis of the normality of the distribution was performed with Shapiro–Wilk test. The homogeneity of variance was analyzed by Hartley's test, Cochran–Cox test, and Bartlett's chi-square test. Mauchley's sphericity test was also performed. From the analysis of variance, it follows that the assumptions of a one-way repeated measures analysis of variance are met in the analyzed groups. In order to verify statistical hypotheses, the level of significance of alpha = 0.05 was assumed. The calculations were carried out in Statistica 12.0 (StatSoft, Poland).

## 3. Results

### 3.1. Demographics and Statistics

The present study included 43 Caucasian patients (17 males and 26 females). The mean age was 39.97 ± 3.78 years. Demographic characteristics of the patients are summarized in Table 3. There were no differences in age or gender between the groups (*p* > 0.05).

Data collected using sEMG and VAS were analyzed using descriptive statistics and briefly presented in Table 4.

**Figure 2:**
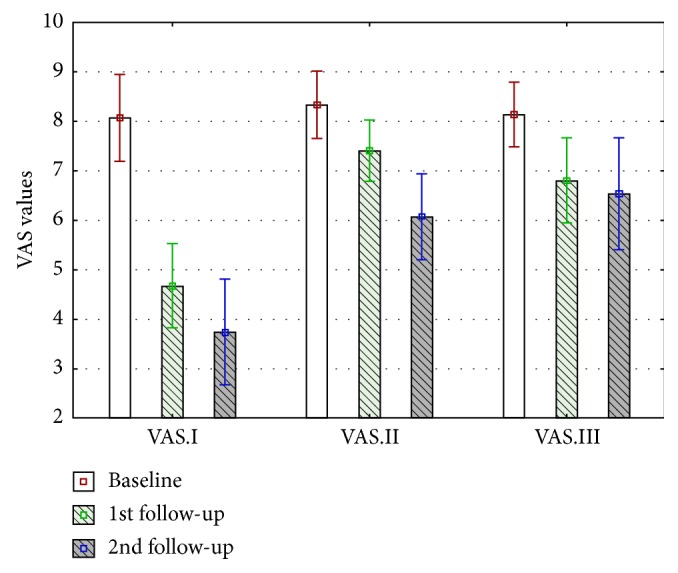
VAS mean value changes in Group I, Group II, and Group III during the trial (days 0, 7, and 14).

**Figure 3:**
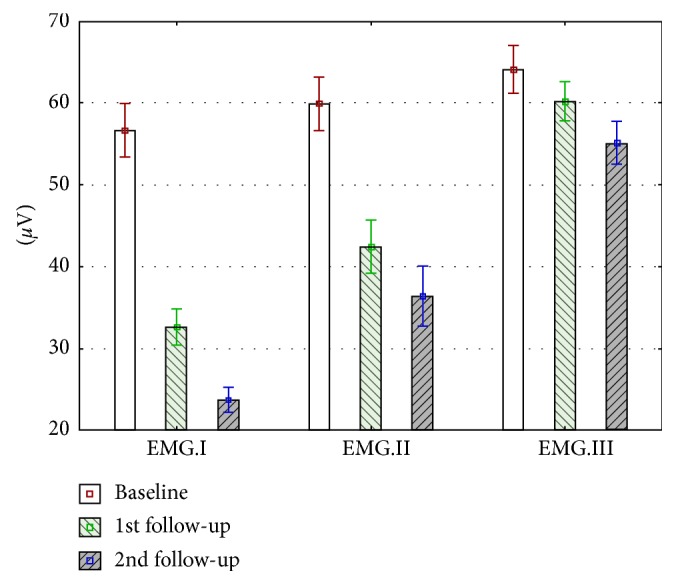
Changes in mean values of superficial electromyographic activity of masseter muscles in Group I, Group II, and Group III during the trial (days 0, 7, and 14).

Collected values for sEMG masseter muscle activity and pain intensity were normally distributed. The statistical analysis showed that the decreases in the mean values of EMG and VAS over time are statistically significant (*p* < 0.001). The mean values and 95% confidence intervals are shown in the Figures 2 and 3.

### 3.2. Primary Treatment Outcome

#### 3.2.1. Evaluation of Masseter Muscle Pain Intensity

Masseter muscle pain intensity was assessed and compared before injection of collagen (VAS.I.1.), lidocaine (VAS.II.1.), and saline (VAS.III.1.) after 7 days (VAS.I.2., VAS. II.2., and VAS. III.2.) and 14 days (VAS.I.3., VAS. II.3., and VAS. III.3.) during baseline and follow-up visits.

Pain intensity reduction was observed in all groups: in Group I, the average pain intensity reduction in VAS scale was 4.3 = 53.75%; in Group II, the average decrease in pain intensity was 2 = 25%; and in Group III, the average value of pain elimination was 1.63 = 20.1% as well (Table 5, Figure 2). Comparing data between measurements performed on days 7 and 14, the authors observed statistically significant pain reduction in all cases, between baseline, 1st follow-up visit, and 2nd follow-up visit (Table 5).

### 3.3. Secondary Treatment Outcome

#### 3.3.1. Evaluation of the Surface Electromyography

Masseter muscle activity was assessed and compared before injection of collagen (EMG.I.1.), lidocaine (EMG.II.1.), and saline (EMG.III.1.) after 7 days (EMG.I.2., EMG. II.2., and EMG. III.2.) and 14 days (EMG.I.3., EMG. II.3., and EMG. III.3.) during follow-up visits. Only rest muscle electromyographic activity was measured in trigger point region on the painful side.

EMG activity of masseter muscles was measured in each group for three times, during baseline and follow-up visits (Figure 3). Mean values for all collected sEMG results are presented in Figure 2. The most significant reduction of sEMG values was observed in Group I (32.9 *μ*V, 59.2%). In Group II, a 23.5 *μ*V (39.3%) reduction was observed. The lowest reduction of sEMG values was noticed in Group III (8.9 *μ*V, 14%) (Table 6). In each group, a statistically significant reduction was observed (*p* < 0.001).

#### 3.3.2. Evaluation of the Surface Electromyography on the Side without Myofascial Pain

Masseter muscle activity was also assessed and compared on the asymptomatic side before injections of collagen (EMG.I.1. NP), lidocaine (EMG.II.1. NP), and saline (EMG.III.1. NP) after 7 days (EMG.I.2. NP, EMG. II.2. NP, and EMG. III.2. NP) and 14 days (EMG.I.3. NP, EMG. II.3. NP, and EMG. III.3. NP) during follow-up visits (Table 6). In 3 subjects, pain was observed bilaterally. In each group, no statistically significant changes of sEMG were observed (*p* > 0.001).

### 3.4. Adverse Effects

Approximately 30 minutes after the injection of collagen into the masseter muscle, patients described pain during movement, edema, and muscle stiffness. After approximately 1 hour, pain symptoms were gone. In a few patients (9 subjects), bruises appeared after the injection, directly at the needle insertion points. These adverse effects were temporary and completely reversible. There were no serious adverse effects during the trial.

## 4. Discussion

Intramuscular injections of collagen, lidocaine, and saline into the trigger points of masseter muscles in the treatment of myofascial pain reduction within masseter muscles varied across study groups in terms of their level of success. The best results were achieved in Group I: maximal reduction of sEMG activity (32.9 *μ*V; 59.2%) and best antinociceptive results (reduction, 4.3; 53.75% on the VAS scale). There are not many research studies analyzing collagen intramuscular injections, besides Milani [34], Yu et al. [35], and Alfieri [36]. These authors stated in their research studies a positive muscle reaction to intramuscular collagen injections, but these studies were not related to orofacial muscle pain.

However, despite the fact that the result is satisfactory, we would like to emphasize that the trial had limitations. The main limitation was the short period of observation of the reduction of pain intensity and the single-blind nature of the trial. Both these limitations resulted in our restricted funding and possibilities of carrying out the trial.

According to the current literature, biomaterial guided regeneration is a new approach for myofascial pain syndrome. This is confirmed by Kuraitis et al. who injected a collagen matrix enhanced with sialyl LewisX (sLeX) to guide skeletal muscle differentiation and regeneration [26]. Muscle tissue damaged by an injected substance has the ability to perform myogenesis and revascularization. We found that satellite cells are active in muscle cell regeneration and collagen VI participates in the activation of satellite cells [17]. The extracellular matrix is a special collagen supply for new myocytes formed in the process of muscle regeneration. The composition of ECM is extremely important for the proper regeneration process to avoid substitution by fibrotic connective tissue, that is, scar production. It is probable that the collagen molecules that were provided by intramuscular injections help to produce an extracellular network that keeps myocytes in their proper positions. The presence of satellite cells in an extracellular matrix is called “a pool” of pluripotential cells for myocyte formation. In this study, the authors noticed better muscle tissue properties and less pathological symptoms after extracellular collagen delivery.

In the clinical trial, we noticed muscle function advancement after collagen intramuscular injections, but Kato et al. found that muscle collagen protein synthesis is not regulated by elevated nutritional or intravenous levels of collagen, but just by mechanical stress [37]. Some authors have observed a better muscle tissue condition and muscle activity decreasing after intramuscular collagen injections. Lawrence and De Luca found a positive correlation between muscle myoelectric signals and the muscle force of the maximal voluntary contraction [38, 39].

In Group II, intramuscular lidocaine injections were performed to decrease pain and to eliminate trigger points. McMillan et al. performed a comparative research between dry needling and procaine injection into the trigger points of masseter muscles in patients with temporomandibular disorders [40]. They concluded that therapy with dry needling and procaine is questionable, because they did not notice any difference in the end point of his study between experimental groups. We found similar results in our study, but in comparison with Group I, lidocaine and dry needling were far less effective.

Antinociceptive results were also observed, but not as successful as in Group I. We can also find some articles about myofascial pain therapy with prolotherapy, which involves the injection of an irritant solution of lidocaine and dextrose into the joint, ligament or painful muscle [41, 42]. Sung et al. identified the correlation between lidocaine concentration and exposure time and tissue cell death [43]. In the future, it would be important to compare anesthetics that are less toxic, for example, ropivacaine. We observed in our research study some effectiveness of injections, with different solutions. We found that we have achieved the best regenerative results with collagen injections, but lidocaine and saline injections also produced pain level decreases as well as sEMG activity decreases. Blasco-Bonora performed a dry-needling technique in masseter muscle trigger points and also achieved an improvement in muscle pain reduction and jaw opening in patients with sleep bruxism [44]. Kalichman and Vulfsons stated in their study that deep dry needling is more effective than superficial dry needling in the therapy of musculoskeletal pain [45]. Masseter muscle lies just underneath the skin, so injections were not very deep (approximately 1.5 cm), but we can call it deep wet needling. Injecting collagen into the trigger point in our opinion may be favorable, not only because of the specific mechanism of action in regenerating muscle tissue, or as a buffer collagen supply, but also as a therapeutic injection. Dry needling and injections into the trigger points have some common points with acupuncture methods [46–49].

It should be noted that a significant effect in terms of reducing sEMG muscle activity and pain intensity was obtained after two injections and the study intervention did not pose a risk of significant adverse effects and high interoperative risk.

## 5. Conclusions

The study confirmed that intramuscular injection of collagen is a more efficient method to reduce myofascial pain within masseter muscles than intramuscular injection of lidocaine. Due to the short observation time, further long-term trials should be conducted.

## Figures and Tables

**Table 1:** Activities of investigators during the trial.

Visit	1 (screening and inclusion)	2 (baseline)	3 (1st follow-up)	4 (2nd follow-up)
Day of the study	**−**	Day 0	Day 7	Day 14

Injection	**−**	+	+	**−**

Measure EMG	**−**	EMG.I.1.	EMG.I.2.	EMG.I.3.
		EMG.II.1.	EMG.II.2.	EMG.II.3.
		EMG.III.1.	EMG.III.2.	EMG.II.3.
		EMG.I.1.NP	EMG.I.2.NP	EMG.I.3.NP
		EMG.II.1.NP	EMG.II.2.NP	EMG.II.3.NP
		EMG.III.1.NP	EMG.III.2.NP	EMG.II.3.NP

Measure VAS	**−**	VAS.I.1.	VAS.I.2.	VAS.I.3.
		VAS.II.1.	VAS.II.2.	VAS.II.3.
		VAS.III.1.	VAS.III.2.	VAS.III.3.

EMG.I.1. = EMG, Group I first measurement; NP = no pain.

**Table 2:** Expected VAS values and measurements.

Observation	Group	Baseline	1st follow-up	2nd follow-up
1	VAS.I	8	5	3
2	VAS.II	8	6	5
3	VAS.III	8	7	6

**Table 3:** Baseline characteristics of 43 patients with MFP within masseter muscles included in the study.

	Group I	Group II	Group III
Male/female, *n*	5/10	5/8	7/8
Age (years)	37.2 ± 4.97	42.8 ± 0.98	40.3 ± 1.18
Duration of myofascial pain (weeks), mean (SD)	30.2 ± 31.48	34.3 ± 29.26	38.3 ± 26.47
Bilateral involvement of myofascial pain (number of patients)	2	1	0

**Table 4:** Descriptive statistics of sEMG and VAS values.

	*N*	Average	Minimum	Maximum	Stand. dev.	One-way repeated measures ANOVA
EMG.I.1. (*μ*V)	15	56.67	47	65	5.95	*p* < 0.001
EMG.I.2. (*μ*V)	15	32.67	28	41	3.85	
EMG.I.3. (*μ*V)	15	23.73	20	29	2.81	

EMG.I.1.NP (*μ*V)	15	34.3	27	45	5.17	*p*=0.344
EMG.I.2.NP (*μ*V)	15	34.6	27	42	4.35	
EMG.I.3.NP (*μ*V)	15	35.2	25	44	5.47	

VAS.I.1.	15	8.07	5	10	1.58	*p* < 0.001
VAS.I.2.	15	4.67	2	8	1.54	
VAS.I.3.	15	3.73	1	7	1.94	

EMG.II.1. (*μ*V)	13	59.07	49	70	4.79	*p* < 0.001
EMG.II.2. (*μ*V)	13	41.20	37	49	3.36	
EMG.II.3. (*μ*V)	13	35.07	29	45	4.40	

EMG.II.1.NP (*μ*V)	13	38.7	29	60	7.3	*p*=0.353
EMG.II.2.NP (*μ*V)	13	39.2	31	55	6.8	
EMG.II.3.NP (*μ*V)	13	37.7	29	52	6.4	

VAS.II.1.	13	8.33	6	10	1.23	*p* < 0.001
VAS.II.2.	13	7.40	5	9	1.12	
VAS.II.3.	13	6.07	4	9	1.58	

EMG.III.1. (*μ*V)	15	64.13	56	72	5.34	*p* < 0.001
EMG.III.2. (*μ*V)	15	60.20	54	69	4.41	
EMG.III.3. (*μ*V)	15	55.27	50	64	4.83	

EMG.III.1.NP (*μ*V)	15	36.6	26	43	8.3	*p*=0.138
EMG.III.2.NP (*μ*V)	15	34	29	41	4.5	
EMG.III.3.NP (*μ*V)	15	36.5	29	42	4.3	

VAS.III.1.	15	8.13	6	10	1.19	*p* < 0.001
VAS.III.2.	15	6.80	4	9	1.57	
VAS.III.3.	15	6.53	3	9	2.03	

**Table 5:** Changes in VAS mean values in Group I, Group II, and Group III after 14 days.

Visit	Group I	Group II	Group III
Baseline	8	8.3	8.13
1st follow-up visit	4.6	7.4	6.8
2nd follow-up visit	3.7	6	6.5
VAS changes	**−4.3**	**−2**	**−1.63**
Percentage VAS changes	**−53.75%**	**−25%**	**−20.1%**

**Table 6:** Changes in EMG mean values in Group I, Group II, and Group III after 14 days.

Visit	Group I (*μ*V)	Group II (*μ*V)	Group III (*μ*V)
*Pain side*			
Baseline	56.6	59.9	64.1
1st follow-up visit	32.6	42.4	60.2
2nd follow-up visit	23.7	36.4	55.2
EMG changes	**−32.9**	**−23.5**	**−8.9**
Percentage EMG changes	**−59.2%**	**−39.3%**	**−14%**

*No pain side*			
Baseline	34.3	38.7	36.6
1st follow-up visit	34.6	39.2	34
2nd follow-up visit	35.2	37.7	36.5
EMG changes	**+0.9**	**−1**	**−0.1**
Percentage EMG changes	**+2.6%**	**−2.5%**	**−0.3%**

## Data Availability

The datasets supporting the conclusions of this article are included within the article. Access to other data will be considered by the corresponding author upon request.
